# Early deaths associated with community-acquired and healthcare-associated bloodstream infections: a population-based study, Finland, 2004 to 2018

**DOI:** 10.2807/1560-7917.ES.2022.27.36.2101067

**Published:** 2022-09-08

**Authors:** Keiju SK Kontula, Kirsi Skogberg, Jukka Ollgren, Asko Järvinen, Outi Lyytikäinen

**Affiliations:** 1 Division of Infectious Diseases, Inflammation Center, Helsinki University Hospital and University of Helsinki, Helsinki, Finland; 2 Department of Health Security, Finnish Institute for Health and Welfare, Helsinki, Finland

**Keywords:** mortality, community-acquired, healthcare-associated, bloodstream infection, population-based, early death, comorbidity

## Abstract

**Background:**

Bloodstream infections (BSI) cause substantial morbidity and mortality.

**Aim:**

We explored the role of causative pathogens and patient characteristics on the outcome of community-acquired (CA) and healthcare-associated (HA) BSI, with particular interest in early death.

**Methods:**

We used national register data to identify all BSI in Finland during 2004–18. We determined the origin of BSI, patients´ underlying comorbidities and deaths within 2 or 30 days from specimen collection. A time-dependent Cox model was applied to evaluate the impact of patient characteristics and causative pathogens on the hazard for death at different time points.

**Results:**

A total of 173,715 BSI were identified; 22,474 (12.9%) were fatal within 30 days and, of these, 6,392 (28.4%) occurred within 2 days (7.9 deaths/100,000 population). The 2-day case fatality rate of HA-BSI was higher than that of CA-BSI (5.4% vs 3.0%). Patients who died within 2 days were older than those alive on day 3 (76 vs 70 years) and had more severe comorbidities. Compared with other BSI, infections leading to death within 2 days were more often polymicrobial (11.8% vs 6.3%) and caused by *Pseudomonas aeruginosa* (6.2% vs 2.0%), fungi (2.9% vs 1.4%) and multidrug-resistant (MDR) pathogens (2.2% vs 1.8%), which were also predictors of death within 2 days in the model.

**Conclusions:**

Overrepresentation of polymicrobial, fungal, *P. aeruginosa* and MDR aetiology among BSI leading to early death is challenging concerning the initial antimicrobial treatment. Our findings highlight the need for active prevention and prompt recognition of BSI and appropriate antimicrobial treatment.

## Introduction

Bloodstream infections (BSI) are a major health concern. According to a systematic review published in 2013, nearly 2 million BSI episodes and 250,000 deaths from BSI were estimated to occur annually in North America and Europe combined [1]. Despite advances in antimicrobial therapy and intensive care, mortality of BSI remains high, with 1-month case fatality rates of ca 10–19% for community-acquired BSI (CA-BSI) and even higher rates, 17–28%, for healthcare-associated BSI (HA-BSI) [2-6].

Previous studies have shown that a marked proportion, approximately one third of BSI-associated deaths occur early, within 2 days after the positive blood culture specimen [7-9]. To our knowledge, only one previous population-based study has reported 2-day case fatality rates of BSI, yet this study presents data from 1992–97 and consists of solely CA-BSI [7]. Possible predisposing factors for early mortality have not been studied; however, delayed diagnosis, inappropriate antimicrobial treatment, and the patient’s overall condition may contribute to an early fatal outcome of BSI. In general, the initial antimicrobial treatment of BSI is challenging since the confirmation of the causative pathogen and its susceptibility are typically not known until the second day after collecting blood cultures. Thus, some BSI-associated deaths occur before the definitive results from the blood cultures are known.

Our recent population-based study from Finland showed a twofold increase in the incidence and mortality of BSI during 2004–18, which is presumably related to ageing of the population and the rising burden of underlying medical conditions [10]. However, the 30-day case fatality rate remained steady over time (12.6–13.0%). In the present study, we used the same national laboratory-based surveillance data linked to other national registers to analyse all BSI in Finland during 2004–18 with the aim of evaluating the role of different factors, such as comorbidity and causative pathogens, on the outcome of BSI of both community and healthcare origin, with focus on early death (≤ 2 days).

## Materials and methods

### Study setting and population

The healthcare system in Finland (population: 5.2 million in 2004 and 5.5 million in 2018 [11]) is organised into 20 healthcare districts, which include a total of five tertiary care hospitals, 15 secondary care hospitals and numerous primary care hospitals. All clinical microbiology laboratories notify bacterial and fungal isolates from blood samples, i.e. BSI, to the National Infectious Disease Register (NIDR). The notifications are reported electronically and include the following information: specimen date, pathogen, patient date of birth, sex, place of residence, and national identity code. Multiple notifications of the same pathogen containing the same identity code, i.e. referring to the same person, are merged into a case if they occur within 3 months of each other.

In this retrospective cohort study, we used NIDR data to identify all BSI in Finland during 2004–18. Only BSI with valid identity codes were included in the study (n = 587 excluded) (flow chart of the data in [10], Figure 1). Duplicate notifications (same specimen date, pathogen, and identity code) were excluded (n = 155). Information on the hospitalisation of the patient, including origin of the infection, i.e. CA-BSI vs HA-BSI, and current and prior (within 1 year) diagnosis codes were obtained by linkage to the National Hospital Discharge Register (HILMO).

### Definitions

BSI was defined as the occurrence of viable bacteria or fungi in the blood evidenced by positive blood cultures. Polymicrobial BSI was defined as isolation of more than one bacterial or fungal species in blood cultures within 2 days.

BSI was classified as healthcare-associated (HA) if the first blood culture was obtained more than 2 days after admission to the hospital or within 2 days of discharge [12]. Patients with a BSI who were transferred from other healthcare facilities, including nursing homes, were also classified as having a HA-BSI. Patients with CA-BSI had not previously been hospitalised and their blood culture specimen was obtained within 2 days of hospital admission.

Comorbid illness was defined by using a validated algorithm for the Charlson comorbidity index (CCI) based on the International Classification of Diseases, 10th revision [13,14]. Three levels of comorbidity were defined by the CCI scores: low (score 0) for patients with no reported underlying diseases included in the CCI, medium (score 1–2) and high (score > 2) [15,16].

### Multidrug-resistant pathogens

The interpretation of susceptibility data of causative pathogens was performed in the clinical microbiology laboratories by using the CLSI standard for samples collected before year 2011 and, afterwards, according to the EUCAST clinical breakpoints [17]. The following bacteria with susceptibility data notified to the NIDR were defined as multidrug-resistant (MDR) pathogens: methicillin-resistant *Staphylococcus aureus* (MRSA) (starting year 1995), vancomycin-resistant *Enterococcus* (VRE) (1995), extended-spectrum beta-lactamase-producing (ESBL) *Escherichia coli* and *Klebsiella pneumoniae* (2008), and carbapenem-resistant Enterobacteriaceae (CRE) (2015). ESBL-*E. coli* and ESBL-*K. pneumoniae* were defined as resistant or intermediately susceptible to third-generation cephalosporins, and CRE as *E. coli, K. pneumoniae* and *Enterobacter* sp. resistant or intermediately susceptible to carbapenems.

### Outcome

The case fatality rate at 2 or 30 days (1 month) after withdrawal of the first blood specimen with a positive culture of a particular patient was determined from the Population Information System by linkage with the identity code. All deaths occurring within 2 days after collection of the specimen yielding the first positive blood culture (day 0) were referred to as early deaths.

### Analyses and statistics

The average annual 2-day and 30-day mortality rates (early and overall deaths/100,000 population, respectively) were calculated according to the total number of deaths and population during 2004–18; population data was retrieved from Statistics Finland [11]. Univariate analysis of categorical variables was done with the chi-squared test, using Yates’s correction or Fisher's exact test, as appropriate. The differences in distributions between continuous variables were tested by the Kruskal-Wallis test. A time-dependent Cox model was applied to evaluate the effects of patient characteristics and causative pathogens on the hazard for death within different time points (within 2 or 3–30 days after the positive blood culture). Data was analysed using SPSS Statistics version 25 (IBM) and Stata 16 (StataCorp).

## Results

### Study population and causative pathogens

During 2004−18, a total of 173,715 BSI among 147,953 patients were identified; 70.9% were CA-BSI and 29.1% HA-BSI (Table 1). Of the patients with HA-BSI, 56.7% were male, whereas no sex difference was noted in CA-BSI. The percentage of persons aged ≥ 70 years and the proportion of patients with high CCI (score > 2) were significantly greater among HA-BSI compared with CA-BSI (52.9% vs 50.3% and 29.3% vs 18.0%, respectively).

**Table 1:** Characteristics of patients with community-acquired and healthcare-associated bloodstream infections, Finland, 2004–2018 (n = 173,715 bloodstream infections)

Characteristics		All BSI n = 173,715		Community-acquired BSI n = 123,232		Healthcare-associated BSI n = 50,483		p value
		n	%	n	%	n	%	
**Sex **								
Male		90,231	51.9	61,619	50.0	28,612	56.7	< 0.001
Female		83,484	48.1	61,613	50.0	21,871	43.3	
**Age (years)**								
Median (range)		70 (0–110)		70 (0–110)		71 (0–104)		< 0.001
Group	< 20	8,943	5.1	6,313	5.1	2,630	5.2	< 0.001
	20–69	76,062	43.8	54,898	44.5	21,164	41.9	
	≥ 70	88,710	51.1	62,021	50.3	26,689	52.9	
**Charlson comorbidity index**								
Median (range)		1 (0–15)		1 (0–15)		2 (0–15)		< 0.001
Score	0	70,328	40.5	57,883	47.0	12,445	24.7	< 0.001
	1–2	66,364	38.2	43,110	35.0	23,254	46.1	
	> 2	37,023	21.3	22,239	18.0	14,784	29.3	
**Causative pathogen**								
**Gram-positive bacteria**		**79,960**	**46.0**	**55,718**	**45.2**	**24,242**	**48.0**	< 0.001^a^
*Staphylococcus aureus*		22,297	12.8	14,596	11.8	7,701	15.3	
Coagulase-negative staphylococci		14,114	8.1	7,763	6.3	6,351	12.6	
Beta-haemolytic streptococci		13,068	7.5	11,427	9.3	1,641	3.3	
*Streptococcus pneumoniae*		11,472	6.6	10,309	8.4	1,163	2.3	
Enterococci		7,348	4.2	3,211	2.6	4,137	8.2	
Viridans streptococci		5,538	3.2	4,121	3.3	1,417	2.8	
Other Gram-positive bacteria		6,123	3.5	4,291	3.5	1,832	3.6	
**Gram-negative bacteria**		**79,520**	**45.8**	**59,455**	**48.2**	**20,065**	**39.7**	
*Escherichia coli*		50,188	28.9	40,103	32.5	10,085	20.0	
*Klebsiella* sp.		9,025	5.2	6,220	5.0	2,805	5.6	
*Pseudomonas aeruginosa*		3,670	2.1	1,752	1.4	1,918	3.8	
*Enterobacter* sp.		3,097	1.8	1,800	1.5	1,297	2.6	
Other Gram-negative bacteria		13,540	7.8	9,580	7.8	3,960	7.8	
**Fungi**		**2,547**	**1.5**	**418**	**0.3**	**2,129**	**4.2**	
*Candida albicans*		1,601	0.9	219	0.2	1,382	2.7	
Non-albicans *Candida*		915	0.5	193	0.2	722	1.4	
Other fungi		31	0.02	6	0.005	25	0.05	
**Other pathogens (not classified)**		**341**	**0.2**	**229**	**0.2**	**112**	**0.2**	
**Polymicrobial BSI**		**11,347**	**6.5**	**7,412**	**6.0**	**3,935**	**7.8**	
**MDR pathogens^b^**		**3,150**	**1.8**	**1,983**	**1.6**	**1,167**	**2.3**	**< 0.001**

BSI: bloodstream infection; MDR: multidrug-resistant.

Differences between community-acquired and healthcare-associated BSI were evaluated with the chi-squared test for categorial variables and with the Kruskal-Wallis test for continuous variables.

^a^ The p value was determined for the differences in the main groups of causative pathogens, i.e. Gram-positive bacteria, Gram-negative bacteria, fungi, other pathogens and polymicrobial BSI.

^b^ MDR pathogens include extended-spectrum beta-lactamase-producing (ESBL) *Escherichia coli* and *Klebsiella pneumoniae,* methicillin-resistant *Staphylococcus aureus* (MRSA), vancomycin-resistant *Enterococcus* (VRE) and carbapenem-resistant Enterobacteriaceae (CRE).

The most common causative pathogens of CA-BSI were *E. coli*, *S. aureus*, beta-haemolytic streptococci, and *Streptococcus pneumoniae*. Among HA-BSI, more *S. aureus*, coagulase-negative staphylococci (CNS) and enterococci were observed, but fewer *E. coli* BSI. Altogether, 3,150 BSI (1.8%) were caused by MDR pathogens (Table 1); 1.2% of all BSI were caused by ESBL-*E. coli* (n = 2,167), 0.1% by ESBL-*K. pneumoniae* (n = 171), 0.3% by MRSA (n = 562), 0.04% by VRE (n = 66), 0.02% by CRE (n = 37), and the rest of MDR pathogens were noted among polymicrobial BSI. About two-thirds (1,641/2,503 BSI; 65.6%) of the BSI caused by ESBL-*E. coli* and ESBL-*K. pneumoniae* and half of the MRSA BSI (286/562 BSI; 50.9%) were community-acquired.

### Case fatality rates at different time points

Of all BSI, 6,392 (3.7%) were fatal within 2 days (7.9 early deaths/100,000 population, range by year: 6.1−10.1) and 22,474 (12.9%) within 30 days (27.9 overall deaths/100,000 population, range by year: 19.5−39.0); among CA-BSI, 30.4% (3,667/12,056) of all deaths occurred within 2 days and among HA-BSI 26.2% (2,725/10,418). The 2-day and 30-day case fatalities were higher for HA-BSI than for CA-BSI (5.4% vs 3.0% and 20.6% vs 9.8%), and slightly higher for males than for females (Table 2). The case fatalities increased with age and with rising CCI; for persons aged ≥ 70 years the case fatalities were over 1.7-fold higher compared with younger and for patients with high CCI over three times greater compared with those with low CCI. Concerning causative pathogens, the highest 2-day and 30-day case fatalities were noted for *P. aeruginosa* (10.8% and 23.5%) and fungi (7.3% and 33.9%), and for polymicrobial BSI (6.7% and 20.6%). For MDR pathogens, the 2-day and 30-day case fatalities were 4.5% and 16.7%, respectively.

**Table 2:** Case fatality rate of bloodstream infections at different time points by patient characteristics, origin of infection and causative pathogens, Finland, 2004–2018 (n = 173,715 bloodstream infections)

Characteristics		All BSI n = 173,715	2-day case fatality		30-day case fatality	
			n	%	n	%
**Sex**						
Male		90,231	3,533	3.9	12,399	13.7
Female		83,484	2,859	3.4	10,075	12.1
**Age group (years)**						
All		173,715	6,392	3.7	22,474	12.9
< 20		8,943	96	1.1	211	2.4
20–69		76,062	2,217	2.9	7,341	9.7
≥ 70		88,710	4,079	4.6	14,922	16.8
**Charlson comorbidity index**						
Score	0	70,328	1,379	2.0	4,163	5.9
	1–2	66,364	2,768	4.2	9,799	14.8
	> 2	37,023	2,245	6.1	8,512	23.0
**Origin of infection**						
Community-acquired BSI		123,232	3,667	3.0	12,056	9.8
Healthcare-associated BSI		50,483	2,725	5.4	10,418	20.6
**Causative pathogen**						
**Gram-positive bacteria**		**79,960**	**2,687**	**3.4**	**10,542**	**13.2**
*Staphylococcus aureus*		22,297	865	3.9	3,845	17.2
Coagulase-negative staphylococci		14,114	286	2.0	1,519	10.8
Beta-haemolytic streptococci		13,068	417	3.2	1,140	8.7
*Streptococcus pneumoniae*		11,472	441	3.8	1,055	9.2
Enterococci		7,348	224	3.0	1,421	19.3
Viridans streptococci		5,538	134	2.4	587	10.6
Other Gram-positive bacteria		6,123	320	5.2	975	16.0
**Gram-negative bacteria**		**79,520**	**2,756**	**3.5**	**8,687**	**10.9**
*Escherichia coli*		50,188	1,260	2.5	4,126	8.2
*Klebsiella* sp.		9,025	343	3.8	1,191	13.2
*Pseudomonas aeruginosa*		3,670	398	10.8	863	23.5
*Enterobacter* sp.		3,097	105	3.4	424	13.7
Other Gram-negative bacteria		13,540	650	4.8	2,083	15.4
**Fungi**		**2,547**	**185**	**7.3**	**864**	**33.9**
*Candida albicans*		1,601	108	6.7	553	34.5
Non-albicans *Candida*		915	76	8.3	304	33.2
Other fungi		31	1	3.2	7	22.6
**Other pathogens (not classified)**		**341**	**8**	**2.3**	**39**	**11.4**
**Polymicrobial BSI**		**11,347**	**756**	**6.7**	**2,342**	**20.6**
**MDR pathogens^a^**		**3,150**	**142**	**4.5**	**526**	**16.7**

BSI: bloodstream infection; MDR: multidrug-resistant.

^a^ MDR pathogens include extended-spectrum beta-lactamase-producing (ESBL) *Escherichia coli* and *Klebsiella pneumoniae,* methicillin-resistant *Staphylococcus aureus* (MRSA), vancomycin-resistant *Enterococcus* (VRE), and carbapenem-resistant Enterobacteriaceae (CRE).

### Predictors of early death

Patients who died early, within 2 days, were older than those who were alive at day 3 (median age: 76 vs 70 years) (Table 3). The proportion of HA-BSI and patients with high CCI were greater in BSI leading to early death compared with those of other BSI (42.6% vs 28.5% and 35.1% vs 20.8%, respectively). *E. coli* was the most common causative pathogen of BSI leading to early death (19.7%), followed by *S. aureus* (13.5%), *S. pneumoniae* (6.9%), beta-haemolytic streptococci (6.5%) and *P. aeruginosa* (6.2%). The 2-day case fatality rates and proportions of fatal episodes by causative pathogens are presented in the Supplement (Supplementary Table S1: all BSI, Supplementary Table S2: CA-BSI and Supplementary Table S3: HA-BSI). Polymicrobial, *P. aeruginosa* and fungal BSI were more common among patients who died early compared with patients who were alive at day 3 (11.8% vs 6.3%, 6.2% vs 2.0% and 2.9% vs 1.4%, respectively), as were also BSI caused by MDR pathogens (2.2% vs 1.8%).

**Table 3:** Predictors of early death caused by bloodstream infection in Finland during 2004–2018 (n = 173,715 bloodstream infections)

Characteristics		Death within 2 days n = 6,392		Alive at day 3 n = 167,323		p value
		n	%	n	%	
**Sex**						
Male		3,533	55.3	86,698	51.8	< 0.001
Female		2,859	44.7	80,625	48.2	
**Age (years)**						
Median (range)		76 (0–103)		70 (0–110)		< 0.001
**Charlson comorbidity index**						
Median (range)		2 (0–14)		1 (0–15)		< 0.001
Score	0	1,379	21.6	68,949	41.2	< 0.001
	1–2	2,768	43.3	63,596	38.0	
	> 2	2,245	35.1	34,778	20.8	
**Origin of infection**						
Community-acquired BSI		3,667	57.4	119,565	71.5	< 0.001
Healthcare-associated		2,725	42.6	47,758	28.5	
**Causative pathogen**						
**Gram-positive bacteria**		**2,687**	**42.0**	**77,273**	**46.1**	< 0.001^a^
*Staphylococcus aureus*		865	13.5	21,432	12.8	
Coagulase-negative staphylococci		286	4.5	13,828	8.3	
Beta-haemolytic streptococci		417	6.5	12,651	7.6	
*Streptococcus pneumoniae*		441	6.9	11,031	6.6	
Enterococci		224	3.5	7,124	4.3	
Viridans streptococci		134	2.1	5,404	3.2	
Other Gram-positive bacteria		320	5.0	5,803	3.5	
**Gram-negative bacteria**		**2,756**	**43.1**	**76,764**	**45.9**	
*Escherichia coli*		1,260	19.7	48,928	29.2	
*Klebsiella* sp.		343	5.4	8,682	5.2	
*Pseudomonas aeruginosa*		398	6.2	3,272	2.0	
*Enterobacter* sp.		105	1.6	2,992	1.8	
Other Gram-negative bacteria		650	10.2	12,890	7.7	
**Fungi**		**185**	**2.9**	**2,362**	**1.4**	
*Candida albicans*		108	1.7	1,493	0.9	
Non-albicans *Candida*		76	1.2	839	0.5	
Other fungi		1	0.02	30	0.02	
**Other pathogens (not classified)**		**8**	**0.1**	**333**	**0.2**	
**Polymicrobial BSI**		**756**	**11.8**	**10,591**	**6.3**	
**MDR pathogens^b^**		**142**	**2.2**	**3,008**	**1.8**	**0.013**

BSI: bloodstream infection; MDR: multidrug-resistant.

Differences between the outcome groups were evaluated with the chi-squared test for categorial variables and with the Kruskal-Wallis test for continuous variables.

^a^ A p value was determined for the differences in the main groups of causative pathogens, i.e. Gram-positive bacteria, Gram-negative bacteria, fungi, other pathogens and polymicrobial BSI.

^b^ MDR pathogens include extended-spectrum beta-lactamase-producing (ESBL) *Escherichia coli* and *Klebsiella pneumoniae,* methicillin-resistant *Staphylococcus aureus* (MRSA), vancomycin-resistant *Enterococcus* (VRE), and carbapenem-resistant Enterobacteriaceae (CRE).

### Trends in bloodstream infections leading to early death

During 2004–18, a slight decrease was noted in the 2-day case fatality rate, from 4.1% (317/7,819) to 3.3% (557/17,009) in all BSI, from 3.3% (174/5,227) to 2.7% (358/13,284) in CA-BSI and from 5.5% (143/2,592) to 5.3% (199/3,725) in HA-BSI. The median age of the BSI patients who died early increased from 73 to 79 years and the percentage of those with high CCI rose from 29% to 39% during 2004–18. The proportion of *E. coli* BSI leading to early death increased from 18.3% (58/317) to 20.8% (116/557) and *S. aureus* BSI from 12.6% (40/317) to 16.5% (92/557), whereas *S. pneumoniae* BSI decreased from 13.2% (42/317) to 3.6% (20/557) during 2004–18. The proportion of ESBL-*E. coli* BSI of all *E. coli* BSI leading to early death rose from 1.4% (1/73) to 11.2% (13/116), whereas the proportion of MRSA BSI of *S. aureus* BSI leading to early death declined slightly, from 2.5% (1/40) to 2.2% (2/92).

### Risk factors for death of community-acquired and healthcare-associated bloodstream infections within 2 or 30 days

To evaluate possible risk factors for BSI fatality, we conducted a time-dependent Cox model demonstrating the effect of patient characteristics and causative pathogens on the hazard for death in two outcome groups, death within 2 or 3–30 days; CA-BSI and HA-BSI were analysed separately. Sex and CCI, together with the most common causative pathogens of all BSI (*E. coli* and *S. aureus*) and those associated with the highest 2-day case fatalities (*P. aeruginosa*, fungi and polymicrobial BSI), were included in the model. A clustering effect of healthcare districts was taken into account by robust standard errors, and calendar year was included as a continuous variable. The effect of age on risk of death was presented by a 10-year increase in age. Increasing age and CCI were risk factors for death in both outcome groups and among both CA-BSI and HA-BSI (Figure); the highest hazard ratio (HR: 3.19) was noted among patients with CA-BSI leading to death within 3–30 days with high CCI (Figure, panel D). Male sex was associated with a slightly higher hazard for death in both outcome groups among CA-BSI (Figure, panels C and D). In both HA-BSI and CA-BSI leading to death within 2 days, the highest hazards for death related to specific pathogens were noted for *P. aeruginosa* (HR: 2.74 and 2.07, respectively) (Figure, panels A and C). For BSI with a fatal outcome within 3–30 days, the highest hazards for death were observed for *S. aureus* in CA-BSI (HR: 1.82) (Figure, panel D) and for fungi in HA-BSI (HR 2.32) (Figure, panel B). The hazard ratios of polymicrobial BSI were similar between the outcome groups (Figure, panels A–D). The hazard ratios were higher for ESBL-*E. coli* compared with non-ESBL-*E. coli* both within 2 or 3–30 days (HR: 1.28–1.54 vs 0.58–0.87) (Figure, panels A–D).

**Figure:**
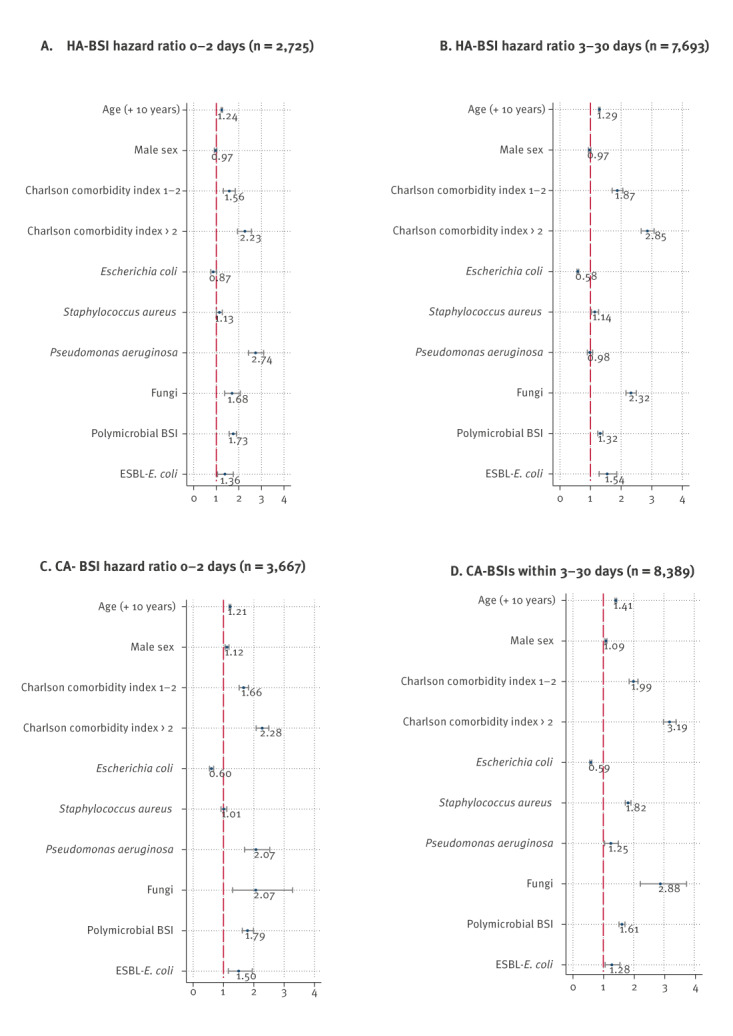
Hazard ratios for death of healthcare-associated bloodstream infections (n = 50,483) and community-acquired bloodstream infections (n = 123,232) according to patient characteristics and causative pathogens in different outcome groups, Finland, 2004–2018

BSI: bloodstream infection; CA-BSI: community-acquired bloodstream infection; ESBL-*E. coli*: extended-spectrum beta-lactamase-producing *Escherichia coli*; HA-BSI: healthcare-associated bloodstream infection.

Black dots indicate hazard ratios and vertical lines signify 95% confidence intervals.

## Discussion

Our population-based study of over 170,000 BSI in Finland during 2004–18 offers a comprehensive overview on the outcome, particularly early death, of both CA-BSI and HA-BSI. The 30-day case-fatality was 12.9%, and nearly one third of these deaths occurred early, within 2 days. We noted higher 2-day and 30-day case fatalities for HA-BSI compared with CA-BSI. Older age and a greater burden of comorbidities were associated with early BSI mortality. BSIs with early fatal outcome were more often polymicrobial and caused by *P. aeruginosa*, fungi and MDR pathogen compared with other BSI.

We conducted the time-dependent Cox model separately for HA-BSI and CA-BSI based on the knowledge that hospitalised patients are older and have more severe underlying conditions than patients who have acquired a BSI in the community. Also, the treatment guidelines for empiric antimicrobial therapy differ between CA-BSI and HA-BSI based on the differences in causative pathogens. Our model allowed direct comparison of hazards for death concerning causative pathogens of BSI in two outcome groups, as the effect of increasing age and burden of comorbidity was similar between the groups. Although *E. coli* and *S. aureus* were the most common causative pathogens of all BSI and of those leading to death within 2 days, they were not predictors of early death. However, *P. aeruginosa* aetiology was distinctly associated with increased risk for early death, whereas polymicrobial and fungal BSI were associated with a fatal outcome both within 2 days and 3–30 days. Polymicrobial BSI accounted for over 10% of all BSI leading to early death, and *P. aeruginosa* and fungal BSI together for over 15% of HA-BSI leading to early death. The empiric antimicrobial therapy of these BSI is a challenge for clinicians, as the lack of antifungal or broad-spectrum coverage may contribute to early fatality. In fact, a previous study showed that inappropriate initial treatment of *P. aeruginosa* BSI was associated with increased hospital mortality [18]. In our former population-based case series of BSI leading to early death in Southern Finland in 2007, empiric antimicrobial therapy was inappropriate in nearly 30% of the BSI; in 12% of CA-BSI and in 50% of HA-BSI [19]. These inappropriately treated BSI leading to early death were mainly caused by intrinsically resistant Gram-negative bacteria (most commonly *P. aeruginosa*). Consistently, previous reports demonstrate that healthcare-associated status in general is a predictor of ineffective empiric antimicrobial treatment of BSI [20] and of 30-day BSI mortality [5,21].

In our study, the 2-day case fatalities for CA-BSI and HA-BSI were 3.0% and 5.4%, respectively; 30.4% of the deaths among CA-BSI and 26.2% among HA-BSI occurred early. In a survey of community-onset BSI from Calgary, Canada during 2003–07, 38% of the deaths occurred by day 2, equalling a 2-day case fatality rate of 4.7% [8], whereas a slightly higher 2-day case fatality rate (7.2%) was presented in an older population-based cohort study of CA-BSI from North Jutland, Denmark, during 1992–97 [7]. The 30-day case fatality rate observed in the present study (12.9%) is comparable to rates in recent population-based reports (12.8–16.9%) [5,22,23]. Our 2-day and 30-day case fatalities were higher for HA-BSI than for CA-BSI. This is in line with findings from a Swedish study spanning from 2000–13 that demonstrated a 30-day case fatality rate of 17.2% for hospital-acquired BSI and 10.6% for community-onset BSI [5]. In our study, the patients who died early had more underlying diseases and were older compared with other BSI patients, which was also noted in our previous case series [19]. In a Danish survey, only 2% of the BSI patients who died early had no predisposing underlying condition, and these patients were older than survivors [24]. Similarly, former studies have shown that rising age and comorbidity are associated with 30-day BSI mortality [5,7,21,25-27].

Overall, the 2-day case fatality rate of BSI in our study decreased slightly during 2004–18, from 4.1% to 3.3%, possibly indicating advancements in recognition and accuracy of empiric antimicrobial treatment of BSI. In a Danish survey of CA-BSI during 1992–97, the 2-day case fatalities for given pathogens were considerably higher compared with those of CA-BSI in our study; for *S. pneumoniae* 9.3% vs 3.6%, respectively, for *S. aureus* 9.0% vs 3.1%, for *E. coli* 6.3% vs 2.0%, and for polymicrobial BSI 10.7% vs 5.9% [7]. The descending overall 2-day case fatality rate noted in the present study might reflect changes in causative pathogens of BSI leading to early death over time, such as the increase in the proportion of *E. coli* BSI but also the decline in *S. pneumoniae* BSI. The observed reduction in *S. pneumoniae* BSI is probably associated with the introduction of the pneumococcal vaccine to the childhood immunisation schedule [28,29]. We observed a distinct ascending trend in the percentage of ESBL-*E. coli* BSI leading to early death during 2004–18. However, the overall proportion of BSI caused by MDR pathogens in our study was low, 2.2% of BSI leading to early death and 1.8% of all BSI, compared with reports from North America and most of Europe excluding the Nordic countries [27,30,31]. Thus, MDR pathogens have not played a substantial role in fatal outcome of BSI in Finland thus far, yet the global threat of antimicrobial resistance may potentially worsen the outcome of BSI in the future.

There are certain limitations in our study. Firstly, we did not have information on possible delays in recognition of the infection and commencement of the treatment, nor data on whether the antimicrobial therapy was appropriate. Delayed and ineffective initial treatment are associated with increased BSI mortality, as demonstrated in previous reports [26,32-34]. In our previous study of BSI leading to early death, the time from symptom onset to administration of antimicrobial therapy was longer in CA-BSI compared with HA-BSI referring to probable delays in seeking medical care [19]. Secondly, we lacked data on detailed clinical features, such as severity of infection (e.g. the respiratory tract as a focus of infection), and information on the role of BSI in the chain of morbid events and on the main cause of death. However, among patients who died early, BSI may have been at least a contributing factor. We did not have data on patients’ underlying medical conditions other than the ones included in the CCI, nor information on a possible do-not-resuscitate (DNR) order, which likely have influenced the outcome. In fact, nearly one third of the BSI patients who died early in our previous case series had either a rapidly fatal underlying condition or a prior DNR order indicating a poor overall condition, and it is probable that most of these deaths were inevitable [19]. Furthermore, we lacked information on behavioural predisposing factors, such as overweight and smoking behaviour, which may affect the outcome [35]. Thirdly, we had limited information on antimicrobial resistance of the causative pathogens, for example, no susceptibility data was available concerning *P. aeruginosa* and *Acinetobacter* sp. Lastly, it is possible that some HA-BSI were inaccurately interpreted as CA-BSI since the hospital discharge register contains data on day surgery only (not all outpatient invasive procedures) and on direct transfers between healthcare facilities. Moreover, the timeframe, blood cultures obtained within 2 days of hospital discharge, for the definition of HA-BSI in the study was quite strict possibly leading to underestimation of HA-BSI.

## Conclusion 

In view of ageing of population in Finland, as in most other industrialised countries, BSI will constitute a major health burden in the future with a risk of fatal outcome, especially among vulnerable patients, elderly people and those with severe comorbidity. In our study, a notable proportion of BSI patients died early, and probably at least some of these deaths were inevitable. However, one fifth of those who died early had no recorded underlying medical condition, which emphasises the importance of rapid recognition of BSI and prompt initiation of adequate antimicrobial treatment according to the origin of the infection. The 2-day case fatality rate of BSI might potentially be used as an indicator of effectiveness of the healthcare system and the treatment chain. The 2-day and 30-day case fatalities were higher for HA-BSI in the present study underlining the need for considerable efforts in prevention of BSI in healthcare facilities. Active surveillance of causative pathogens and their resistance trends is beneficial when composing local guidelines for empiric antimicrobial therapy of BSI. Although the proportion of BSI caused by MDR pathogens was low in our study, the growing problem of antimicrobial resistance causes concern worldwide. Further studies are needed to evaluate the impact of the COVID-19 pandemic on the incidence, outcome, and causative pathogens of BSI, particularly on the occurrence of resistant pathogens.

## Supplementary Data

111000 Supplement
