# Supplementary material for: Early deaths associated with community-acquired and healthcare-associated bloodstream infections: a population-based study, Finland, 2004 to 2018
Source: Euro Surveill. 2022 Sep 8;27(36):2101067. doi: 10.2807/1560-7917.ES.2022.27.36.2101067 (PMC9461309; doi:10.2807/1560-7917.ES.2022.27.36.2101067)
Supplement: Supplement [file 21-01067_KONTULA_SUPPLEMENT.pdf]

Supplementary Material

This supplementary material is hosted by *Eurosurveillance* as supporting information alongside the article ´Early deaths associated with community-acquired and healthcare-associated bloodstream infections: a population-based study, Finland, 2004–2018´, on behalf of the authors, who remain responsible for the accuracy and appropriateness of the content. The same standards for ethics, copyright, attributions and permissions as for the article apply. Supplements are not edited by *Eurosurveillance* and the journal is not responsible for the maintenance of any links or email addresses provided therein.

Table S1. Two-day case fatality rate of bloodstream infections and proportion of fatal episodes by causative pathogens, Finland, 2004–2018 (n=173,715)

Pathogen	Number of all BSIs caused by the pathogen	Number of deaths within 2 days caused by the pathogen	2-day case fatality rate, %	Proportion of fatal episodes within 2 days caused by the pathogen, %
Gram-positive bacteria	79 960	2687	3.4	42.0
Staphylococcus aureus	22 297	865	3.9	13.5
Coagulase-negative staphylococci	14 114	286	2.0	4.5
β-hemolytic streptococci	13 068	417	3.2	6.5
Streptococcus pneumoniae	11 472	441	3.8	6.9
Enterococci	7348	224	3.0	3.5
Viridans streptococci	5538	134	2.4	2.1
Other Gram-positive bacteria	6123	320	5.2	5.0
Gram-negative bacteria	79 520	2756	3.5	43.1
Escherichia coli	50 188	1260	2.5	19.7
Klebsiella sp.	9025	343	3.8	5.4
Pseudomonas aeruginosa	3670	398	10.8	6.2
Enterobacter sp.	3097	105	3.4	1.6
Other Gram-negative bacteria	13 540	650	4.8	10.2
Fungi	2547	185	7.3	2.9
Candida albicans	1601	108	6.7	1.7
Non-albicans *Candida*	915	76	8.3	1.2
Other fungi	31	1	3.2	0.02
Other pathogens (not classified)	341	8	2.3	0.1

Polymicrobial BSI	11 347	756	6.7	11.8
**Total**	**173 715**	**6392**	**3.7**	**100**
MDR pathogens	3150	142	4.5	2.2

Abbreviations: BSI, bloodstream infection; MDR, multidrug-resistant.

Table S2. Two-day case fatality rate of community-acquired bloodstream infections and proportion of fatal episodes by causative pathogens, Finland, 2004–2018 (n=123,232)

Pathogen	Number of CA-BSIs caused by the pathogen	Number of deaths within 2 days caused by the pathogen	2-day case fatality rate, %	Proportion of fatal episodes within 2 days caused by the pathogen, %
Gram-positive bacteria	55 718	1640	2.9	44.7
Staphylococcus aureus	14 596	454	3.1	12.4
Coagulase-negative staphylococci	7763	124	1.6	3.4
β-hemolytic streptococci	11 427	338	3.0	9.2
Streptococcus pneumoniae	10 309	370	3.6	10.1
Enterococci	3211	51	1.6	1.4
Viridans streptococci	4121	100	2.4	2.7
Other Gram-positive bacteria	4291	203	4.7	5.5
Gram-negative bacteria	59 455	1554	2.6	42.4
Escherichia coli	40 103	800	2.0	21.8
Klebsiella sp.	6220	183	2.9	5.0
Pseudomonas aeruginosa	1752	140	8.0	3.8
Enterobacter sp.	1800	40	2.2	1.1
Other Gram-negative bacteria	9580	391	4.1	10.7
Fungi	418	28	6.7	0.8
Candida albicans	219	19	8.7	0.5
Non-albicans *Candida*	193	9	4.7	0.2
Other fungi	6	0	0	0
Other pathogens (not classified)	229	4	1.7	0.1
Polymicrobial BSI	7412	441	5.9	12.0
**Total**	**123 232**	**3667**	**3.0**	**100**
MDR pathogens	1983	73	3.7	2.0

Abbreviations: CA-BSI, community-acquired bloodstream infection; BSI, bloodstream infection; MDR, multidrug-resistant.

Table S3. Two-day case fatality rate of healthcare-associated bloodstream infections and proportion of fatal episodes by causative pathogens, Finland, 2004–2018 (n=50,483)

**Pathogen**	**Number of HA-BSIs caused by the pathogen**	**Number of deaths within 2 days caused by the pathogen**	**2-day case fatality rate, %**	**Proportion of fatal episodes within 2 days caused by the pathogen, %**
Gram-positive bacteria	24 242	1047	4.3	38.4
Staphylococcus aureus	7701	411	5.3	15.1
Coagulase-negative staphylococci	6351	162	2.6	5.9
β-hemolytic streptococci	1641	79	4.8	2.9
Streptococcus pneumoniae	1163	71	6.1	2.6
Enterococci	4137	173	4.2	6.3
Viridans streptococci	1417	34	2.4	1.2
Other Gram-positive bacteria	1832	117	6.4	4.3
Gram-negative bacteria	20 065	1202	6	44.1
Escherichia coli	10 085	460	4.6	16.9
Klebsiella sp.	2805	160	5.7	5.9
Pseudomonas aeruginosa	1918	258	13.5	9.5
Enterobacter sp.	1297	65	5.0	2.4
Other Gram-negative bacteria	3960	259	6.5	9.5
Fungi	2129	157	7.4	5.8
Candida albicans	1382	89	6.4	3.3
Non-albicans *Candida*	722	67	9.3	2.5
Other fungi	25	1	4	0.04
Other pathogens (not classified)	112	4	3.6	0.1
Polymicrobial BSI	3935	315	8.0	11.6
**Total**	**50 483**	**2725**	**5.4**	**100**
MDR pathogens	1167	69	5.9	2.5

Abbreviations: HA-BSI, healthcare-associated bloodstream infection; BSI, bloodstream infection; MDR, multidrug-resistant.
